# Algorithm to Correct Measurement Offsets Introduced by Inactive Elements of Transducer Arrays in Ultrasonic Flow Metering

**DOI:** 10.3390/s22239317

**Published:** 2022-11-30

**Authors:** Jack Massaad, Paul L. M. J. van Neer, Douwe M. van Willigen, Michiel A. P. Pertijs, Nicolaas de Jong, Martin D. Verweij

**Affiliations:** 1 Laboratory of Medical Imaging, Department of Imaging Physics, Delft University of Technology, Lorentzweg 1, 2628 CJ Delft, The Netherlands; 2 KROHNE New Technologies B.V., Kerkeplaat 12, 3313 LC Dordrecht, The Netherlands; 3 Netherlands Organisation for Applied Scientific Research (TNO), Oude Waalsdorperweg 63, 2597 AK The Hague, The Netherlands; 4 Electronic Instrumentation Laboratory, Department of Microelectronics, Delft University of Technology, Mekelweg 4, 2628 CD Delft, The Netherlands; 5 Erasmus MC, Thorax Center, Department of Biomedical Engineering, Doctor Molewaterplein 40, 3015 GD Rotterdam, The Netherlands

**Keywords:** guided waves, transducer arrays, ultrasonic flow meter

## Abstract

Ultrasonic flow meters (UFMs) based on transducer arrays offer several advantages. With electronic beam steering, it is possible to tune the steering angle of the beam for optimal signal-tonoise ratio (SNR) upon reception. Moreover, multiple beams can be generated to propagate through different travel paths, covering a wider section of the flow profile. Furthermore, in a clamp-on configuration, UFMs based on transducer arrays can perform self-calibration. In this manner, userinput is minimized and measurement repeatability is increased. In practice, transducer array elements may break down. This could happen due to aging, exposure to rough environments, and/or rough mechanical contact. As a consequence of inactive array elements, the measured transit time difference contains two offsets. One offset originates from non-uniform spatial sampling of the generated wavefield. Another offset originates from the ill-defined beam propagating through a travel path different from the intended one. In this paper, an algorithm is proposed that corrects for both of these offsets. The algorithm also performs a filtering operation in the frequency-wavenumber domain of all spurious (i.e., flow-insensitive) wave modes. The advantage of implementing the proposed algorithm is demonstrated on simulations and measurements, showing improved accuracy and precision of the transit time differences compared to the values obtained when the algorithm is not applied. The proposed algorithm can be implemented in both in-line and clamp-on configuration of UFMs based on transducer arrays.

## 1. Introduction

Ultrasonic flow meters (UFMs) are cheap and reliable sensors with a broad linearity and relatively small footprint on the pipeline compared to flow sensors based on other physical phenomena [1,2]. Current UFMs consist of one or more pairs of single element transducers, and flow is measured using the transit time difference method: a first transducer, located upstream to the flow direction, generates an ultrasonic wave that propagates through the liquid and is recorded by the second transducer, located downstream to the flow direction. Similarly, this second transducer also generates an ultrasonic wave that propagates and is ultimately recorded by the upstream transducer. Due to the non-reciprocity of the flow (i.e., it moves in only one direction), there is a transit time difference Δt between the recorded upstream and downstream waves, which is proportional to the flow speed $v_{f}$ [1,3,4]:

(1) $$v_{f}=\frac{c_{0}^{2}\Delta t}{2L\cos(\theta_{liquid})}.$$

where $c_{0}$ represents the sound speed of the flowing liquid, *L* represents the path length of the acoustic beam, and $\theta_{liquid}$ represents the angle of propagation of the acoustic beam relative to the pipe axis. UFMs exist in two configurations: inline and clamp-on. In the former configuration, the transducers are in direct contact with the liquid inside the pipe, while in the latter configuration, the transducers are located on the the outside surface of the pipe wall [1,5].

There are several advantages in using transducer arrays in UFMs compared to using single element transducers: electronic beam steering [6,7] allows to properly align the direction of propagation of the generated acoustic beams with the location of the receiving transducer array. In [8], this was achieved by monitoring the amplitude of the receiving array while changing the beam steering angle of the acoustic beam in fine steps. Ultimately, a peak amplitude value is recorded, resulting in an optimal signal-to-noise ratio (SNR) of the recorded signals. Moreover, transducer arrays allow to generate several acoustic beams propagating through different acoustic paths. In the context of UFMs, this can be leveraged to obtain a more complete sample of the flow profile and ultimately in more accurate flow measurements [9]. With clamp-on UFMs, acoustic properties of the pipe and the liquid are needed to compute the flow speed. In current sensors, these parameters are introduced as a priori information by the user. Different techniques have been proposed to measure these parameters [10,11,12,13,14,15]. These techniques are carried out on solid materials, such as pipes, submerged in water, which is not the typical setting for UFMs. Furthermore, these techniques are based on the generation of guided waves in the solid material, and require some a priori information regarding the solid material and the liquid. Transducer arraybased UFMs have the potential to self-calibrate, i.e., to measure the properties of the pipe and the liquid [8,16]. The techniques in [8,16] are also based on generating guided waves in the pipe wall, however, they are more suited for UFMs since they do not require the pipe to be submerged nor any a priori information regarding the pipe or the liquid, rendering input from the operator unnecessary and thus increasing measurement repeatability.

During ultrasonic flow metering, spurious wave modes are usually present. These are coherent signals associated with acoustic waves that propagate through media and travel paths different to that of the desired (flow-sensitive) wave mode. In the case of a clamp-on UFM configuration, these are usually guided waves propagating through the pipe wall and/or reflections from obstacles along the pipeline, such as flanges, bends or cracks in the pipe wall. These spurious wave modes could potentially overlap in the timespace (t−x) domain with the desired wave mode, and since they cannot be averaged out, they ultimately introduce an offset in the measured transit time difference, and thus in the measured flow speed [17]. Array-based UFMs offer a solution to this issue: data recorded in the t−x domain may be taken to the frequency-wavenumber ($f-k_{x}$) domain [18], where the flow sensitive wave mode can be identified, isolated from spurious wave modes, and taken back to the t−x domain to finally compute a more accurate transit time difference. An important requirement to implement this filtering operation properly is to have uniform spatio-temporal sampling of the generated wavefield.

Through the lifetime of a transducer array, the individual piezo elements may break or become inactive or non-responsive due to aging, fabrication flaws (i.e., mechanical and electrical heterogeneities along the piezo-elements) or mechanical damage. This is similar to the degradation observed in medical imaging applications [19,20,21]. Given a mechanical load applied on an array (e.g., pre-stress during installation or by accidental hits during operation), the transfer function (impulse response) of the individual transducer array elements may change with time and differ per element. Thus, some elements are more likely to become inactive than others. In the context of transducer array-based UFMs, inactive elements cause the filtering operation mentioned above to perform sub-optimally since there is no longer a uniform spatial sampling of the acoustic wavefield. Furthermore, inactive elements result in the generation of acoustic beams that propagate through a travel path different than the one intended with a fully working array, thus resulting in an extra offset introduced in the measured transit time difference. When individual array elements are inactive, it may be unpractical to replace them. Thus, the effects mentioned above need to be accounted for.

In this paper, an algorithm that corrects for the measurement offsets introduced by inactive elements of array-based UFMs is proposed. With this algorithm, it becomes possible to keep measuring flow using transducer arrays with inactive elements. Moreover, the proposed algorithm helps in minimizing SNR deterioration caused by the inactive elements. The algorithm performs three major operations: first, a reference signal is selected and properly assigned as the response of the inactive element(s); second, a filtering operation of the desired (flow-sensitive) wave mode is performed in the $f-k_{x}$ domain; and third, the measured transit times of the acoustic beams generated by faulty transducer arrays, which propagated through a different travel path, are corrected. The rest of the paper is structured as follows. Section 2 describes the measurement offsets introduced by inactive transducer array elements in the context of array-based UFMs. Section 3 describes the proposed algorithm to correct for the measurement offsets described in Section 2. Section 4 shows the implementation of the proposed algorithm on simulated data and its validity on experimental data. Section 5 presents a discussion of the results. Section 6 summarizes the conclusions.

## 2. Measurement Offsets Introduced by Inactive Transducer Array Elements

### 2.1. On the Shape of the Beam

**Figure 1:**
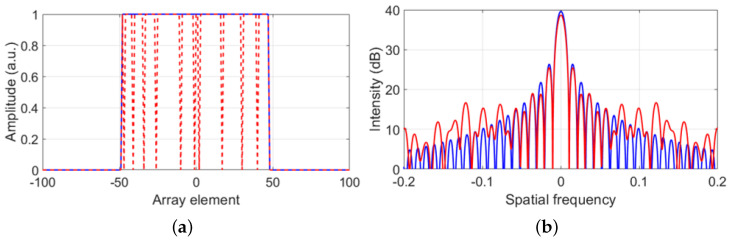
(**a**) Spatial aperture of a 96−element transducer array with fully working elements (in blue) and 10 inactive elements (in red). (**b**) Fourier transform of the spatial apertures shown in (**a**).

The 2D aperture of a transducer array consisting of *N* elements in a 3D space can be represented in 1D by a rectangular window (see blue curve in Figure 1a):

(2) $$h\left(x\right)=\left\{\begin{array}{cc} 1 & \forall x\in [-N/2,N/2]\\ 0 & \mathrm{otherwise}. \end{array}\right.$$

In the Fourier domain, the aperture of a transducer array (of Equation (2)) is described by a sinc function [22], described by a main beam lobe and side lobes (see blue curve in Figure 1b).

In the context of fully-working array-based UFMs (blue curve in Figure 1a), the generated wavefield is recorded with a uniform spatial sampling. Thus, the Fourier transform of the recorded beam is described by a function with a predictable shape (see blue curve in Figure 1b). Moreover, most of the flow-sensitive information is located on the main lobe of the received beams. A filter capable of keeping only the main lobe, therefore filtering-out all spurious information, can be realized and implemented in a straightforward manner, ultimately resulting in a more accurate transit time difference compare to the value obtained without performing the filtering operation.

When flow measurements are performed with transducer arrays with inactive elements (red curve in Figure 1a), the generated wavefield is recorded with non-uniform spatial sampling. As a result, the Fourier transform of this non-uniform spatial aperture is no longer a sinc function. Instead, it presents a non-uniform distribution of the amplitudes of the side lobes (see red curve in Figure 1b). In addition to increasing noise, this effect becomes a problem when higher-than-expected side lobes associated to the acoustic beams of spurious wave modes overlap with the main lobe of the flow-sensitive acoustic beam and cannot be filtered-out, resulting in a measured transit time difference with an offset from the correct value. Thus, to suppress this offset, it is important to ensure, in post-processing, uniform spatial sampling of the recorded wavefield.

There are a few methods that could be implemented to realize a representative “virtual” measurement for an inactive element, thus achieving uniform spatial sampling of the wavefield. Some of these are implemented in seismic data analysis, where it is usually the case that a large number of receivers is used and some of them may be damaged, or non-responsive, or simply, by certain geographical limitations or legal restrictions, could not be placed at the desired location. Therefore, trace interpolation techniques are usually implemented [23,24,25,26,27]. However, careful attention is needed in the implementation of these techniques on faulty transducer arrays used for ultrasonic flow measurements since it is not straightforward to know what the ultimate effects of these techniques are on the phase of the interpolated traces, which may end up adding an extra offset to the measured transit time difference. Another method is to assign the measured signal with the highest amplitude as a “virtual” measurement of the inactive elements. However, during the selection process of this signal, it also needs to be considered how much of its amplitude corresponds to spurious wave modes, which may not be straightforward to implement practically since it may not always be known how much of the recorded amplitudes is associated to spurious wave modes. A more practical approach is to pre-store the impulse response of each transducer array element after its fabrication, and in case of failure, use this pre-recorded signal, with a proper phase, as a “virtual” measurement of the inactive element.

### 2.2. On the Travel Path

**Figure 2:**
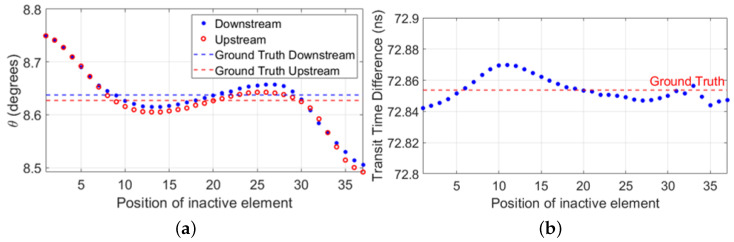
Simulation results obtained using FIELD II [28,29] to generate acoustic beams in water steered at an angle of $\theta =8.6{}^{\circ }$ relative to the normal of the surface of a 37-element transducer array. The flow effect was included by defining the sound speed of the medium as $c=1480\;m/s\mp v_{f}\cos\left(8.6{}^{\circ }\right)$, with $v_{f}=0.5\;m/s$ being the flow speed, and where ∓ defined the acoustic wave propagating upstream and downstream, respectively. The beams were measured in the far field. (**a**) Location, relative to the surface of the transducer array, of the peak amplitude of each illgenerated beam as a function of the position at which the inactive element is located on the array. The dashed horizontal lines show the ground truth locations, i.e., those obtained with fully working arrays. (**b**) Transit time difference offset introduced by the distorted travel paths described by the ill-defined beams. The dashed horizontal line indicates the ground truth transit time difference, i.e., the one obtained with fully working arrays.

With a transducer array, a steered acoustic beam is generated by applying a unique time delay on the excitation signal of each array element. A transducer array with fullyworking elements can generate an acoustic beam steered under an angle θ relative to the normal of the surface of the transducer array. A line can be drawn between the point location of the peak amplitude of the beam in the far field and the center point of the array aperture. The angle between this line and the normal of the surface of the transducer array is equal to the beam steering angle θ of the acoustic beam. However, when the same time delays are implemented on the same array, but with inactive elements, an ill-defined beam is generated. In the far field, the line drawn between the location of the peak amplitude of this beam and the center point of the array aperture makes an angle, relative to the normal of the surface of the array, different to the intended angle θ (see Figure 2a). This means that the ill-generated beam propagates through a different travel path and therefore has a different transit time compared to the beam generated by a fully-working array. In the context of array-based UFMs, this translates into an offset introduced on the transit time difference (see Figure 2b). Therefore, to suppress this offset, it is necessary to correct for this difference in transit time. The corrected transit time, $T_{corrected}$ may be achieved in transmission by adjusting the steering angle of the ill-generated beam, or in reception by phase-shifting the measured signal by:

(3) $$\Delta t_{correction}=T_{measured}-T_{gt},$$

where $T_{gt}$ represents the ground truth transit time, i.e., the transit time of the acoustic beam generated by a fully-working transducer array. In the context of array-based UFMs, $T_{gt}$ depends on the properties of the liquid and the pipe, as well as on the flow speed. However, it can be obtained from measurements performed with fully-working arrays, or it can be calculated relatively fast using Rayleigh’s second integral.

## 3. Algorithm

**Figure 3:**
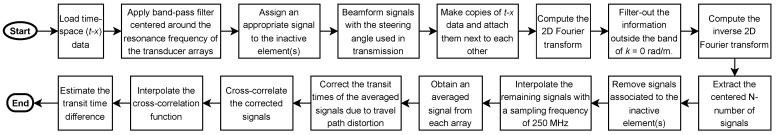
Flowchart of the designed algorithm to correct for the measurement offsets introduced by inactive (non-functioning/broken/damaged) elements during transducer array-based ultrasonic flow metering.

A flowchart of the proposed algorithm to correct for the measurement offsets introduced by inactive elements during transducer array-based ultrasonic flow measurements is shown in Figure 3. Each array is assumed to have the same total number of elements *N*. The operations are described in detail below.

To reduce random noise, a 5th-order Butterworth filter is applied on the recorded signals. The center frequency of this filter corresponds to the center (resonance) frequency of the transducer array. Next, to avoid the measurement offset associated to the effect shown in Figure 1b, a representative signal is assigned as a “virtual” measurement of the inactive elements, naturally with the appropriate phase for each one of them. In this case, the waveform recorded with the highest measured amplitude is assigned as the representative signal. At the position of each inactive element, the proper phase of the representative signal is extrapolated from the phase of signals measured by neighbouring working elements, taking also into account the known phase shift of the input signal for that particular inactive element that would have resulted in the generation of the beam steered at the intended direction. Afterwards, the t−x data of each array is beam-formed, i.e., the individual signals are phase-shifted by the same time delays used in transmission to generate a steered acoustic beam. As a result, the signals measured by all array elements report the same transit time of the flow-sensitive wave mode. Due to the finite aperture of the arrays, to increase the resolution of the measured wave modes in the wavenumber dimension, the t−x data of each array is copied a few times along the spatial dimension. Then, the 2D Fourier transform is computed. As a consequence of beam-forming, the information associated to the flow-sensitive wave mode in the $f-k_{x}$ domain is located at the wavenumber $k_{x}=0\;\mathrm{rad}/m$ for all temporal frequency components. To remove all information associated to spurious wave modes, a simple filter in the $f-k_{x}$ domain is designed. For all temporal frequency components, the filter has a value of 1 in the wavenumber range $\left[-\frac{2\pi }{\Delta x};\frac{2\pi }{\Delta x}\right]$, with Δx being the array pitch, and a value of 0 outside of this wavenumber range. The filtering operation consists of multiplying the designed filter with the computed 2D Fourier transform of the t−x data. Then, the inverse 2D Fourier transform is computed to obtain the filtered signals in the t−x domain. Given the copy operation of the t−x data performed earlier, per array, the center *N* number of filtered signals is kept. Furthermore, to maintain reciprocity, the filtered signals associated with the inactive elements are discarded for the rest of the analysis. Subsequently, the remaining filtered signals are interpolated with a sampling frequency of 250MHz. From these, an averaged signal is obtained per array. Now, the transit time of each averaged signal is corrected due to the different travel path covered by each ill-generated beam. This correction is achieved by phase-shifting the averaged signals by the factor $\Delta t_{correction}$ shown in Equation (3). Cross-correlation between both corrected averaged signals is performed. Then, the cross-correlation function is interpolated around the peak amplitude, from which ultimately a more accurate transit time difference is obtained.

## 4. Validation of the Algorithm

Simulations and measurements were performed to validate the algorithm proposed in the previous section. Two P4-1 phased array probes (Koninklijke Philips N.V., Eindhoven, NL) were considered, and the liquid medium was water.

### 4.1. Simulation

The software FIELD II [28,29] was used to generate the simulated wavefields.

#### 4.1.1. Settings

As source, a P4-1 phased array probe (Number of array elements: 96, pitch: 0.295mm, elevation: 17mm, center frequency: 1MHz, bandwidth: 1–4MHz) was defined. The spatial domain along the azimuth (*x*) and depth (*z*) direction of the transducer array was discretized as dx=dz=0.295mm (i.e., equal to the array pitch), and the maximum propagation depth was defined to be $z_{max}=160\;\mathrm{mm}$. A 3-cycle Gaussian-apodized sine function with a center frequency of $f_{0}=2.25\;\mathrm{MHz}$ was used as excitation signal. The length of the geometry along the azimuth was defined long enough so that, at $z_{max}$, it was possible to simulate the acoustic pressure at the location of the 96 elements of the receiving array centered around the peak amplitude of the propagated beam. The sound speed of water was set to $c_{0}=1480\;m/s$.

Two wave modes were simulated: a flow-sensitive wave mode and a spurious wave mode. For the flow-sensitive wave mode, two simulations were performed: one for the wavefield propagating upstream to the flow, and another one for the wavefield propagating downstream to the flow. Considering the sound speed $c_{0}$, in both cases, a steered acoustic beam was generated under an angle of $\theta =20{}^{\circ }$. The flow speed $v_{f}$ was taken into account by defining the final sound speed of the medium to be $c=c_{0}\mp v_{f}\cos\left(\theta \right)$ for the upstream (−) and downstream (+) propagating beam.

The spurious wave mode was simulated in a similar way to the flow-sensitive wave mode. However, it was steered under an angle $\theta =15{}^{\circ }$ and in the opposite direction to the flow-sensitive wave mode. Furthermore, the spurious wave mode was simulated to be flow-insensitive, i.e., $v_{f}=0\;m/s$, and to have an amplitude 10dB higher than the amplitude of the flow-sensitive wave mode.

**Figure 4:**
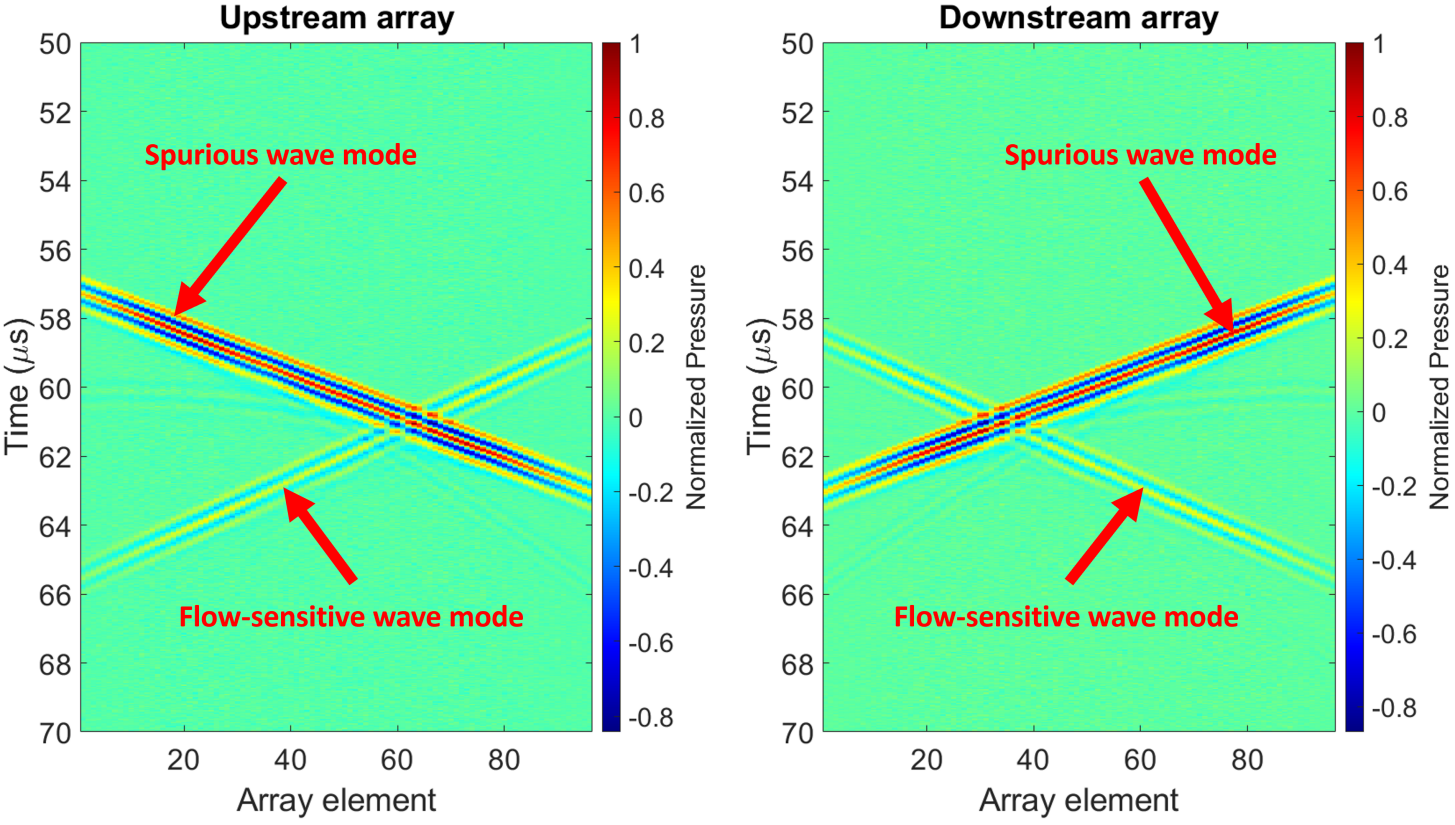
Synthetic pressure wavefield in water generated by simulated apertures of P4−1 probes in the context of array-based UFMs. SNR=30dB per array element. The wavefields were generated using FIELD II [28,29].

Random noise was added to the recorded signals to achieve, per array element, a signal-to-noise ratio (SNR) of 30dB, which is a realistic value for existing transducer arrays. Figure 4 shows the simulated wave modes, and it is representative of a potential scenario of a transducer array-based UFM in a clamp-on configuration, in which a flow-sensitive wave mode may overlap, upon reception, with a spurious wave mode that propagated through a different travel path (e.g., a reflection from an obstacle in the pipeline or a guided wave propagating through the pipe wall).

Inactive transducer array elements were simulated in a straightforward manner. In transmission, an inactive element was assigned an apodization weight of 0, while in reception, the signal was zeroed-out. Two main scenarios of inactive elements were considered: random and grouped locations along the array aperture. The zero-flow case, i.e., $v_{f}=0\;m/s$, was considered.

#### 4.1.2. Results

The robustness and importance of implementing the proposed algorithm is already reflected after analyzing wavefields generated and recorded by fully-working arrays, such as those shown in Figure 4. Considering 50 “virtual” noisy simulated wavefields, and just doing beam-forming, obtaining an averaged signal for each array and crosscorrelating them, a mean transit time difference of Δt=12.1ps with a standard deviation of σ=431.3ps was obtained. In contrast, when implementing the proposed algorithm, a mean transit time difference of Δt=0.93ps with a standard deviation of σ=14.6ps was obtained, i.e., more than an order of magnitude lower for both values.

**Figure 5:**
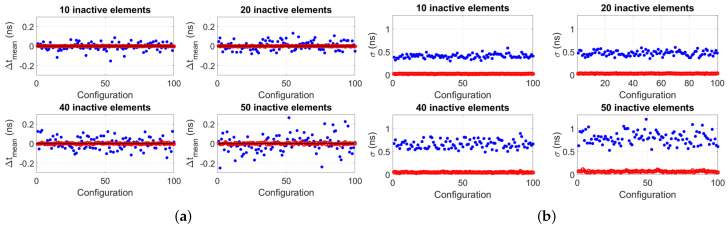
Simulation results of (**a**) mean transit time difference for 100 random configurations for the cases of a total of 10, 20, 40 and 50 inactive array elements situated at random locations along the aperture of 96−elements arrays. Per configuration, a data point represents the mean of 50 “virtual” transit time difference values obtained from 50 simulated noisy wavefields. The blue dots represent the values obtained without implementing the proposed algorithm (i.e., after just beamforming, obtaining and averaged signal per array and cross-correlating them to obtain the transit time difference), and the red circles represent the values obtained after implementing the proposed algorithm. The horizontal black line represents the value obtained without inactive elements, i.e., $\Delta t_{mean}=-0.93\;\mathrm{ps}$. (**b**) Standard deviation of the $\Delta t_{mean}$ value of each configuration reported in (**a**). Each data point represents the standard deviation of 50 “virtual” transit time difference values obtained per configuration.

When simulating random locations of inactive elements, four cases were considered: 10, 20, 40 and 50 inactive elements. Per case, 100 different configurations of the locations of the inactive elements were considered. Moreover, per configuration of inactive elements, 50 noisy virtual wavefields were obtained and processed using the proposed algorithm to obtain 50 transit time difference values, from which a mean value $\Delta t_{mean}$ and standard deviation σ were obtained. Results are summarized in Figure 5.

**Figure 6:**
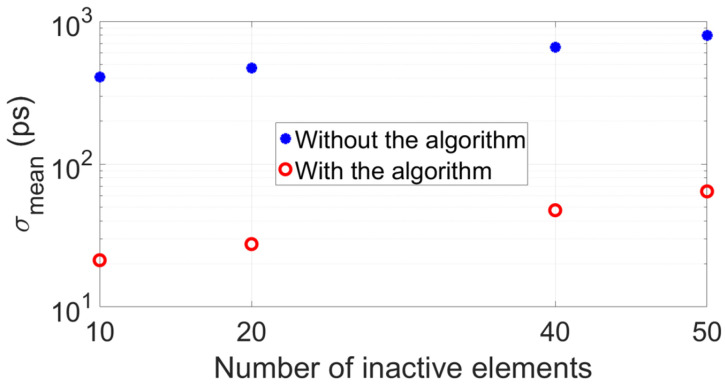
Mean value of the distributions of standard deviation shown in Figure 5b, which were obtained from simulation results.

Figure 5 shows the clear advantage of implementing the proposed algorithm: both accuracy ($\Delta t_{mean}$) and precision (σ) of the transit time difference $\Delta t_{mean}$ improve compared to when the algorithm is not implemented. Figure 6 reports the mean value of the standard deviations of each plot of Figure 5b, $\sigma_{mean}$, showing that this value increases as a function of the number of inactive elements, as expected. However, due to the random locations of the inactive elements, there is a nonlinear relation between the number of remaining active elements and signal SNR, thus, $\sigma_{mean}$ does not worsen with the standard rate of the square root of the number of remaining active elements.

**Figure 7:**
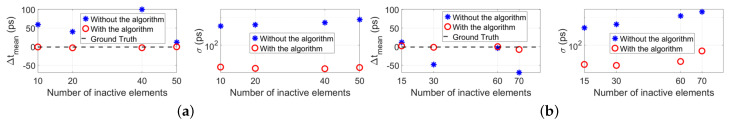
Mean and standard deviation of the transit time difference obtained when considering groups of inactive elements located (**a**) at the center of the aperture of the arrays, and (**b**) at the center and both edges of the aperture of the arrays (i.e., 1/3 of the total number of inactive elements at each location). Per number of inactive elements, a data point of $\Delta t_{mean}$ represents the mean of 50 “virtual” transit time differences values obtained from 50 simulated noisy wavefields, and a data point of σ represents the standard deviation of those 50 “virtual” transit time differences values. The dashed black line on the graphs for $\Delta t_{mean}$ represents the value obtained without inactive elements, i.e., $\Delta t_{mean}=-0.93\;\mathrm{ps}$.

The proposed algorithm was also tested considering grouped locations of inactive transducer array elements. Figure 7a shows the results when considering 10, 20 40 and 50 inactive elements located at the center of the aperture of the arrays. Similarly, Figure 7b shows the results when considering 15, 30, 60 and 70 inactive elements located at the center and both edges (i.e., 1/3 of the total number of inactive elements at each location) of the aperture of the arrays.

In Figure 7, the reported standard deviation are comparable to those reported in Figure 5. At the same time, values of $\Delta t_{mean}$ obtained after implementing the proposed algorithm show a great improvement compared to those obtained without implementing the proposed algorithm. At the same time, it can be seen in Figure 7b that, towards a large number of inactive elements, $\Delta t_{mean}$ starts to deviate more from the ground truth value, and also that σ starts to increase rapidly. These trends are expected due to the increasing effect of non-uniform spatial sampling explained in Section 2.1.

### 4.2. Experiment

To validate the proposed algorithm experimentally, measurements were taken on a custom-build clamp-on ultrasonic flow metering setup.

#### 4.2.1. Setup

**Figure 8:**
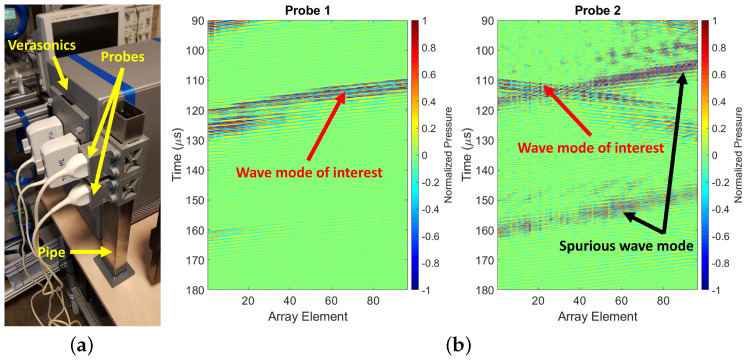
(**a**) Setup to carry out measurements on which to test the proposed algorithm. The probes are two P4−1 transducer arrays clamp-on on the outside of a square stainless steel pipe filled with water. (**b**) Typical measured wavefield. Measurements were carried out at zero-flow conditions.

Two P4-1 transducer array probes were placed on the outside surface of a square stainless steel pipe (wall thickness: 1mm; inner diameter: 40mm; compressional and shear bulk wave sound speeds: $c_{L}=5920\;m/s$ and $c_{T}=3141\;m/s$, respectively, and density: $\rho =7980\;\mathrm{kg}/m^{3}$). The pipe was capped on one side and left open on the other side. The pipe was placed vertically on a table, with some foam material between the table and the capped side of the pipe to minimize acoustic coupling between them. The pipe was left to vibrate freely. The center-to-center distance between both transducer arrays was 80mm, and the pipe was filled with water ($\rho =1000\;\mathrm{kg}/m^{3}$; $c_{L}=1480\;m/s$), see Figure 8a. A Verasonics Vantage 256 system (Verasonics Inc., Kirkland, WA, USA) was used to drive the transducers and record the data. The experiment was done under zero-flow conditions. A 1-cycle square pulse was used as excitation on all transducer array elements. On the pipe wall, a steered acoustic beam was generated under an angle of $\theta_{steel}=50{}^{\circ }$. As a consequence, the shear wave in the pipe wall refracted into the liquid as a compressional wave at an angle of $\theta_{water}=21{}^{\circ }$. The positions of the transducers along the pipe wall ensured the recording of the wave mode of interest, i.e., the compressional wave propagating in the liquid, after two reflections from the opposite side of the pipe (i.e., a w-shape travel path inside the pipe), as well as the recording of spurious wave modes, including those associated with reflected guided waves from one of the open ends of the pipe wall, which also overlapped in the t−x domain with the wave mode of interest (see Figure 8b). This configuration is representative of the simulation setting of Figure 4 and thus of a real case scenario.

#### 4.2.2. Results

**Figure 9:**
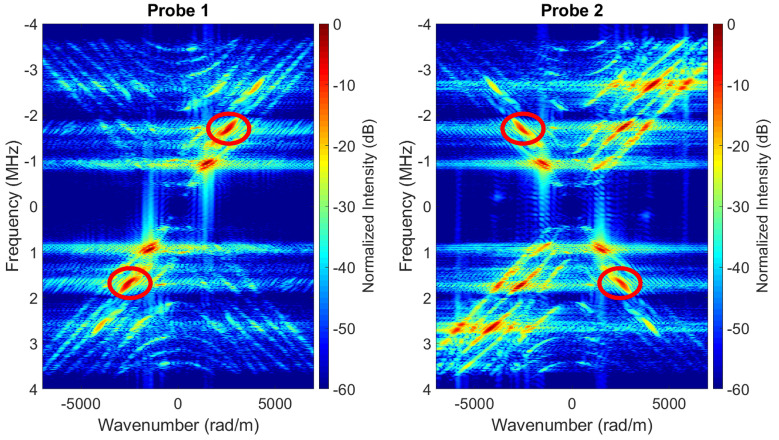
2D Fourier transform of the spatio-temporal data shown in Figure 8b. The red circles frame the bandwidth of energy associated to the wave mode of interest. All other bandwidths of energy are associated to spurious wave modes.

The 2D Fourier transforms of the spatio-temporal wavefields shown in Figure 8b, which were measured using fully-working arrays, are shown in Figure 9, where the different recorded wave modes can be observed. For Probe 2 (right-side image of Figure 9), the red circles in quadrants I and III frame the bandwidths of energy associated with the wave mode of interest. This mode shows amplitudes which are approximately 10dB lower than the amplitudes associated with spurious wave modes. Other bandwidths of energy located within these quadrants are associated with spurious wave modes that propagated in the same direction as the wave mode of interest but through different travel paths. On the other hand, the bandwidths of energy located in quadrants II and IV are associated to spurious wave modes that propagate in opposite direction to the wave mode of interest, which resulted from reflections of the propagating wavefield at the open end of the pipe (i.e., from the steel-air interface). For Probe 1, the location of the different wave modes described above is mirrored relative to Probe 2.

The electrical circuit of the Verasonics machine is designed to drive different standard medical probes, thus, it is not perfectly matched to the electronics of each probe. Also, in practice, it is common for there to be variations of impulse response between the individual transducer array elements. In the context of UFMs, these issues act in detriment of the reciprocity of the system. Therefore, at zero-flow conditions, the transit time difference obtained after implementing the proposed algorithm was not zero, but instead was $\Delta t_{mean}=-0.76\;\mathrm{ns}$.

**Figure 10:**
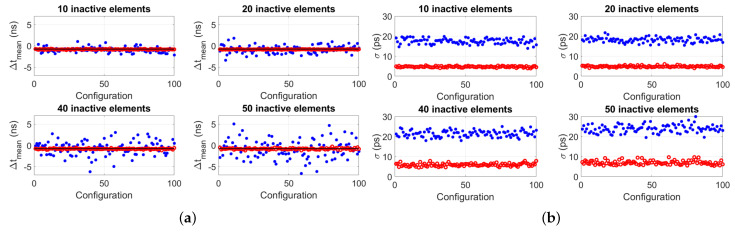
Measurement results of (**a**) mean transit time difference for 100 random configurations for the cases of a total of 10, 20, 40 and 50 inactive array elements situated at random locations along the aperture of 96−elements arrays. Per configuration, a data point represents the mean of 50 transit time difference measurements carried out. The blue dots represent the values obtained without implementing the proposed algorithm (i.e., after just beam-forming, obtaining and averaged signal per array and cross-correlating them to obtain the transit time difference), and the red circles represent the values obtained after implementing the proposed algorithm. The horizontal black line represents the value obtained without inactive elements, i.e., $\Delta t_{mean}=-0.76\;\mathrm{ns}$. (**b**) Standard deviation of the $\Delta t_{mean}$ value of each configuration reported in (**a**). Each data point represents the standard deviation of 50 transit time difference measurements carried out per configuration.

**Figure 11:**
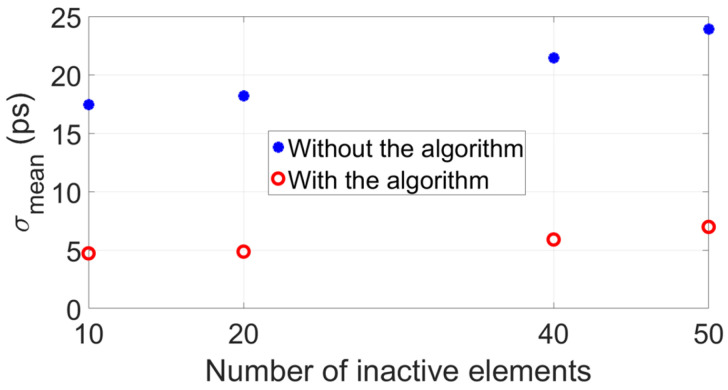
Mean value of the distributions of standard deviation shown in Figure 10b, which were obtained from measurement results.

Analogous to the simulations, the same configurations of 10, 20, 40 and 50 inactive array elements located at random locations along the aperture of the arrays were considered for measurements. Per configuration of inactive elements, 50 measurements of the transit time difference were taken, from which a mean value $\Delta t_{mean}$ and standard deviation σ was obtained. Results are shown in Figure 10, and they clearly show the improved accuracy and precision of the measured $\Delta t_{mean}$ when implementing the algorithm in contrast to when it is not implemented. Furthermore, Figure 11 reports the mean value of the standard deviations of each panel of Figure 10b, $\sigma_{mean}$. These values are lower compared to those reported in Figure 6, which was expected because the Verasonics system used to drive the transducer arrays is optimized to electrically match the P4-1 probes used in the experiments. In fact, for these probes, SNR≈60dB per transducer array element, which is higher than the SNR=30dB value assumed in simulations.

## 5. Discussion

**Figure 12:**
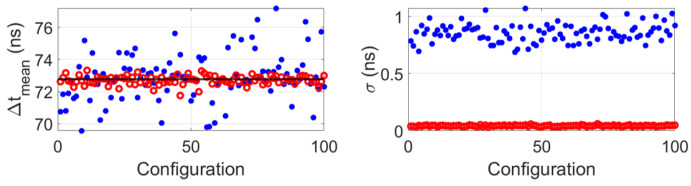
Simulation results of mean (**left**) and standard deviation (**right**) of the transit time difference for 100 random configuration for the case of 10 inactive array elements situated at random locations along the aperture of 96− elements arrays and considering a flow speed of $v_{f}=0.5\;m/s$. Blue dots represent the values obtained without implementing the proposed algorithm (i.e., after just beam-forming, obtaining and averaged signal per array and cross-correlating them to obtain the transit time difference), and the red circles represent the values obtained after implementing the proposed algorithm. The horizontal black line represents the value obtained without inactive elements, i.e., $\Delta t_{mean}=72.78\;\mathrm{ns}$.

Although the proposed algorithm was proven to work on both simulated and measured data at zero flow conditions, simulation results considering a flow speed of $v_{f}=0.5\;m/s$ proved that it should also perform well at flow conditions (see Figure 12). Similarly to previous results, the accuracy of the obtained transit times differences improves compared to when the algorithm is not implemented. Moreover, the mean value of the standard deviations obtained after implementing the proposed algorithm was $\sigma_{mean}=0.04\;\mathrm{ns}$, which is approximately 21x lower than $\sigma_{mean}=0.86\;\mathrm{ns}$, which was the value obtained without implementing the proposed algorithm.

The entire measurement system of an UFM based on transducer arrays could report a high noise level. However, noise may be filtered out provided that its frequency bandwidth does not overlap with that of the transducer arrays. In this manner, the effectiveness of the proposed algorithm is not compromised. However, in practice, both bandwidths tend to overlap, and from experience it should be guaranteed that measurements are acquired with a minimum SNR of 20dB to obtain a useful flow measurement [9]. Ultimately, this also becomes a boundary condition for the effectiveness of the proposed algorithm. Furthermore, another limit is reached when the flow-sensitive wave mode and a spurious wave mode overlap completely in the spatio-temporal domain. In this scenario, both wave modes are also located within the same bandwidth of energy in the $f-k_{x}$ domain, and it would not be possible to filter-out the spurious wave mode. Due to the beam steering capability of transducer arrays, this scenario can be avoided by simply steering the flow-sensitive wave mode under a different angle. On the other hand, in practice, spurious wave modes are usually associated to dispersive guided waves that propagate within the pipe wall. Therefore, depending on how dispersive the propagating frequency components of the spurious wave mode are, some of them may arrive under a slightly different angle compared to the arrival angle of the flow-sensitive wave mode in the $f-k_{x}$ domain. Therefore, it should be possible to filter them out using the proposed algorithm. Furthermore, if some techniques to suppress, in transmission, the excitation of guided waves in the pipe wall [17] are implemented, the remaining frequency components of the spurious wave mode could be recorded with low-enough amplitude to still achieve the desired measurement accuracy.

Given a transducer array with a certain total number of elements *N* and aperture length, it may be possible to discard a certain number of elements in a random manner, while keeping the same aperture length, to achieve a beam profile (in terms of main lobe width and amplitude level of the first few side lobes) similar to that of the *N*-element array. In the context of transducer array-based UFMs, this sparse distribution of active transducer array elements would still introduce the offset in the measured transit time difference discussed in Section 2.1, but it would be minimal compared to the offset introduced by other sparse distributions. The major advantage of achieving the appropriate sparse distribution of active elements would be a reduction in the total number of electronic channels, which would simplify the overall system, including the electronic architecture, resulting in a cheaper sensor at the cost of SNR.

Finally, although the proposed algorithm was tested on simulated and measured data representative in the context of transducer array-based clamp-on UFMs, it could also be implemented on data associated to transducer array-based in-line UFMs.

## 6. Conclusions

In this paper, an algorithm has been proposed to correct for the measurement offsets introduced by inactive array elements in ultrasonic flow metering based on transducer arrays. The algorithm performs three major operations: ensuring uniform spatial sampling of the measured wavefield; filtering of spurious wave modes; and correction of the measured transit times due to a distorted travel path covered by the acoustic beam generated by the faulty arrays. Simulations and measurements haven been carried out considering two 96-element transducer arrays with 10, 20, 40 and 50 inactive elements. The proposed algorithm was implemented on both datasets and it has reported an improvement in both accuracy (by approximately a factor of 13x) and precision (by approximately a factor of 29x and 5x for simulations and measurements, respectively) of the transit time difference.

## Data Availability

Not applicable.

## Abbreviations

The following abbreviations are used in this manuscript:

UFMs	Ultrasonic flow meters
SNR	Signal-to-noise ratio
t-x	time-space
f-k_*x*_	frequency-wavenumber
